# Comparison of Three types of Tooth Brushes on Plaque and Gingival Indices: A Randomized Clinical Trial

**DOI:** 10.2174/1874210601711010126

**Published:** 2017-02-28

**Authors:** Amir Moeintaghavi, Naser Sargolzaie, Mehrnoosh Rostampour, Sara Sarvari, Sanaz Kargozar, Shideh Gharaei

**Affiliations:** 1 Mashhad Dental School, Mashhad University of Medical Sciences, Mashhad, Islamic Republic of Iran; 2 Qom Dental School, Qom University of Medical Sciences - Oral and Maxillofacial Radiology, Qom, Islamic Republic of Iran

**Keywords:** Tooth brushing, Gingivitis, Dental plaque index, Pulsar, CrossAction, Butler

## Abstract

**Objective:**

To compare clinical results of three types of manual tooth brushes on plaque removal efficacy and gingivitis.

**Method:**

This study is a single blind randomized trial with crossover design which involved 30 periodontaly healthy individuals. Professional plaque removal and oral hygiene instruction were performed for all the participants in the first step of our study followed by asking them to avoid brushing for 2 days. Thereafter plaque and gingivitis scores were measured using plaque and gingival indices (PI and GI). Then subjects were instructed to use Pulsar tooth brush for a two-week period and then, GI and PI indices were assessed again. After passing one-week period for wash out, subjects didn't brush for 2 days and indices were recorded again. The same procedure was done for CrossAction, and Butler 411 tooth brushes respectively and at the end of the study these variables were analyzed using SPSS software ver.16. Repeated measurement ANOVA test was used to compare the scores between different brushes.

**Result:**

Finding of this study reveals that using all three types of tooth brushes resulted in significant plaque and gingivitis reduction compared to baseline levels. Pulsar tooth brush was significantly more effective in diminishing PI and GI than Butler tooth brush (p=0.044 and 0.031 respectively).

**Conclusion:**

According to our findings all 3 types of tooth brushes are effective in reduction of plaque and gingivitis and this reduction is significantly greater for Pulsar tooth brush compared to Butler and CrossAction tooth brushes.

## INTRODUCTION

Dental caries and periodontal diseases are among common human illnesses. Researches in the United States of America manifest 47% of 30-year-old adults and older suffering from periodontitis, while 70% of 65-year-old adults illustrate the signs of this disease [1].

It is speculated that the main etiology of periodontal diseases such as gingivitis and periodontitis is the interaction between human immune system and bacteria of dental plaque. Dental caries and periodontal disease can be prevented and well-controlled by sufficient mechanical removal of dental plaque [2]. Otherwise, the biofilm causes gingival inflammation and in severe conditions results in periodontitis and attachment loss [3]. Tooth brushing is utilized as the most widely accepted technique for plaque elimination among adults and children [3-5].

There are numerous factors that affect sufficiency of tooth brushing including frequency, duration, dexterity, tooth brush design and technique. The first three factors are mostly dependent on individual's motivation and behavior and hardly promoted by oral hygiene instructions, though, novel designs evaluated in short intervals may probably increase motivation and sensory skill as well as proper access to the hard-reaching areas [3].

To obtain a promotion in oral hygiene, manufacturers have tried to present an efficacious tooth brush that can remove substantial amount of plaque independently to technique [6].

**Fig. (1):**
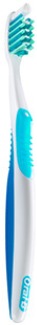
Oral-B CrossAction (Oral B^®^, Procter and Gamble Co., USA).

An example of these ingenious products is Oral-B CrossAction tooth brush (Oral B^®^, Procter and Gamble Co., USA). This brush features bristles that attack plaque in tight space from the right angle (an optimal 18°) cleaning those areas better than straight bristles. Crisscross pattern of bristles have been positioned in opposing directions. These flexible and straightened bristles, actively penetrate between the teeth, where plaque builds up easily, and along the gum lines to lift it out and sweep it away. Soft gum stimulators, positioned on both sides of the brush head, gently massage the gum. Carefully polished end-round bristles are gentle on enamel and gum [7, 8] (Fig. **1**).

**Fig. (2):**
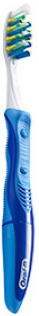
Oral-B^®^ Pulsar™.

Among significant characteristics of Oral-B^®^ Pulsar™, as another paradigm of novel tooth brush, are pulsing by means of the battery in the handle and its bristles that pivot back and forth [8]. Sensitive split head adjusts to the contours of teeth to clean hard-to-reach places while moderating the amount of pressure applies to teeth and gums [9, 10] (Fig. **2**).

**Fig. (3):**
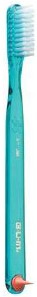
Butler 411(GUM^®^, Sunsatarinc., Chicago, USA).

Despite the new-designed brushes, bristles tufts of Butler 411 (GUM^®^, Sunsatarinc., Chicago, USA) are all the same length and are positioned in three rows which are placed perpendicular to the handle. This is a soft, multi-tufted tooth brush with a head measuring 21 mm in length and 6.5 mm in width (Fig. **3**).

Even though in recent years a large number of long-term studies about manual tooth brushes with a novel design have been conducted, their results have been somewhat inconclusive [11]. This incoordination may be because of various methods and designs of studies [6].

The aim of this study was clinical comparison of effect of three types of manual tooth brushes including CrossAction, Pulsar and Butler on plaque removal and gingivitis.

## MATERIALS AND METHODS

This study was a single blind randomized trial with crossover design which involved 30 periodontally healthy individuals.

All oral examinations were performed at periodontal department, Faculty of dentistry, Mashhad University of Medical Science, Iran. The study was approved by Ethical Committee and written informed consent was taken from all patients and volunteers who wanted to take part in our research. Also, the study protocol has been registered in the clinical trial.gov with this registration number: NCT02412358.

This clinical trial study was carried out on 30 volunteers among sixth-year-dental students (Male: 2, Female: 28, mean age: 23.5±0.7). The inclusion criteria were having at least five teeth in each quadrant; no partial denture; no orthodontic banding or retention wires; no oral lesions or sites with probing pockets depth ≥ 5 mm and routine use of a manual tooth brush. The individuals recruited for this study comprised those who did not have any systematic disease which would influence the course of periodontal diseases or the response to the treatment and also they were not smokers.

Prior to the first experiment, a dental hygienist gave a professional prophylaxis, where plaque and calculus were removed and the teeth were polished. Before the initiation of the study, Plaque Index (PI) and gingival index (GI) were measured with the PI [12] and GI indices [13, 14].

The patients were asked not to brush their teeth for 2 days and then their plaque and gingival indices were recorded and one brush (Pulsar brush, Oral B CrossAction or Butler 411) was given to them for consumption. They were asked to brush twice daily and two minutes for each time by Bass technique. After two weeks they were called and the plaque and gingival indices were recorded again. After a week as wash out period, again they were asked to refrain from brushing for 2 days after which their GI and PI were recorded. Second brush was administered and its two-weeks results were registered. This approach has been repeated for the third one and at the end GI and PI changes for each brush head were compared. The order of tooth brush types was determined randomly.

The mean and standard deviation were calculated for each data and analyzed using SPSS software Ver.16. Normal distribution of data was examined by means of Kolmogorov-Smirnov test. Repeated measurement ANOVA test was used to compare the scores between different brushes. To analyze the PI and GI score changes for each tooth brush Paired sample T- test was used.

## RESULTS

30 dental students (male: 2, female: 28) were enrolled in this study and all completed the study. The mean age of participants was 23.5±0.7.

The mean plaque index in the group used Pulsar tooth brush, changed from 1.55± 0.51 to 0.66±0.44 after two weeks. In CrossAction group it decreased from 1.63±0.44 to 0.55±0.6 after study period. It is worth noting that the mean plaque index in Butler 411 group was 0.92±0.59 at starting and decreased to 0.39±0.44 at the end. The results indicated that plaque index changed significantly for each tooth brush (Table **1**). Gingival index (GI) scores at base line were as follows: 1.18±0.5 (Pulsar), 0.9±0.45 (CrossAction) and 0.73±0.51 (Butler 411) and decreased to 0.65±038, 0.62±0.54 and 0.45±0.44 respectively. These results reveal significant changes in GI before and after using each of three tooth brushes (Table **2**). Paired sample T test showed that GI and PI change in each group of study was statistically significant (Tables **1** and **2**). Tables **3** and **4** show the differences between groups for PI and GI respectively.

## DISCUSSION

Tooth brushing is the most acceptable used-at-home tooth cleaning procedure. Several studies reveal a significant difference between various tooth brushes, while others do not illustrate distinct superiority for any one. Such incoordination could be because of veracity in study design [6,15-21] rather than manifesting reliable clinical consequences.

This Crossover trial has been performed for a two weeks period on 30 dental students. Dental students were examined as the study group in order to eliminate the effect of elements which are related to motivation and behavior habits including dexterity, frequency and duration of tooth brushing. Also, crossover design was used to omit or reduce the interfering variables such as brushing manner and other factors like nutritional habits.

Using the PI needs the minimum utilities including a light resource, a mirror, a probe and explorer. Moreover it is easy and fast [12]. In order to evaluate gingivitis, Gingival Index is used. GI has been suggested since 1963 as a method to evaluate the severity and rate of gingivitis in an individual or a group of people [12-14].

The result of this investigation revealed that Butler 411, CrossAction and Pulsar tooth brushes all appraised significant decrease in mean PI and GI scores (p-value <0.001). In addition, a significant difference between pulsar and butler tooth brush was demonstrated in PI and GI reduction (p-value 0.031, p-value 0.044 respectively).

Warren *et al*. in their study described high percentage of plaque removal by pulsar from the proximal surfaces in comparison with Colgate 360 and Oral-B Advantage Plus [3].

In a study by Sharma *et al*. in 2006, Pulsar tooth brush removed significantly more plaque than Oral-B advantage plus 40 from all areas of the mouth. Additionally, Pulsar tooth brush reduced more marginal and proximal plaque compare to the mentioned tooth brush [22].

Whereas, the result of Birang *et al*. demonstrated that there is no significant difference in plaque reduction between Pulsar, CrossAction and classic. In this study plaque index was recorded in two phases, before and after brushing with Bass technique for two minutes [23].This active observation on the process of brushing may act as a contributing factor and is opposite of the definition of a proper tooth brush.

Moreover, due to our findings CrossAction does not show any nobility among mentioned tooth brushes which is parallel to the results of Cronin *et al*.’s research, in which no significant advantage was observed for CrossAction in compare to battery-powered Actibrush brush in terms of gingivitis reduction and plaque index [24]. This fact is in conflict with results of studies such as Sharma *et al.*, William *et al.* and Huan *et al*. all of which have shown superior plaque removal by the Oral-B CrossAction brush relative to other manual tooth brushes [25-27]. Sharma *et al*. reevaluated their patients after 3 months but in the present study the indices were recorded after two weeks. This might be one of the reasons that justify the mentioned difference between two studies.

It is worth noting that as the samples of our study were all dental students with great oral hygiene consideration, they may not respect the protocol of the study not to brush for 2 days before using each tooth brush. Besides, pulsar tooth brush was the first brush prescribed. These may be the reasons of why there is no significant difference between Butler and CrossAction.

## CONCLUSION

Mechanical plaque control is the most important step in the prevention of periodontal diseases. The present study showed that the plaque and gingival indices will significantly decrease after brushing with each of tooth brush types: CrossAction, Pulsar, and Butler 411. However, those indices were diminished further after brushing with the Pulsar tooth brush.

## Figures and Tables

**Table 1:** Comparison of mean PI score before and after treatment for each of brushes.

Mean ±SD	Before treatment	After treatment	T	P value
pulsar	1.55 ± 0.51	0.66 ± 0.44	7.6	<0.001
CrossAction	1.63 ± 0.44	0.55 ± 0.6	4.9	<0.001
Butler	0.92 ± 0.59	0.39 ± 0.44	4.9	<0.001

**Table 2:** Comparison of mean GI score before and after treatment for each of brushes.

Mean ±SD	Before treatment	After treatment	T	p-value
pulsar	1.18 ± 0.5	0.65± 0.38	5.2	<0.001
CrossAction	0.9 ± 0.45	0.62 ±0.54	3.7	<0.001
butler	0.73 ± 0.51	0.45 ± 0.44	3.8	<0.001

**Table 3:** Comparison of PI reduction differences between groups.

	Mean± SE*	CI^	p-value
CrossAction, pulsar	- 0.28 ± 0.15	(-0.61,0.039)	0.082
Butler, pulsar	-0.36 ± 0.16	(-0.69,-0.035)	0.031
Butler, crossAction	-0.076 ± 0.11	(-0.31,0.16)	0.51

*Std.error, ^Confidence interval.

**Table 4:** Comparison of GI reduction differences between groups.

	Mean ±SD*	CI^	P value
CrossAction, pulsar	- 0.25 ± 0.14	(-0.54,0.047)	0.09
Butler, pulsar	-0.26 ± 0.12	(-0.51,-0.008)	0.044
Butler, crossAction	-0.01 ± 0.074	(-0.16, 0.14)	0.89

*standard deviation, ^confidence interval.
